# Probiotics Mediate Intestinal Microbiome and Microbiota-Derived Metabolites Regulating the Growth and Immunity of Rainbow Trout (Oncorhynchus mykiss)

**DOI:** 10.1128/spectrum.03980-22

**Published:** 2023-03-14

**Authors:** Chunyan Zhao, Xianhui Men, Yongji Dang, Yangen Zhou, Yichao Ren

**Affiliations:** a School of Marine Science and Engineering, Qingdao Agricultural University, Qingdao, Shandong, China; b Key Laboratory of Mariculture, Ministry of Education, Ocean University of China, Qingdao, Shandong, China; University of Minnesota Twin Cities

**Keywords:** rainbow trout, probiotics, gut microbiota, metabolites, immunity, transcriptome

## Abstract

Emerging evidence confirms using probiotics in promoting growth and immunity of farmed fish. However, the molecular mechanisms underlying the host-microbiome interactions mediated by probiotics are not fully understood. In this study, we used rainbow trout (Oncorhynchus mykiss) as a model to investigate the internal mechanisms of host-microbiome interactions influenced by two probiotic bacteria, Bacillus velezensis and Lactobacillus sakei. We carried out experiments, including intestinal histology, serum physiology, and transcriptome and combined intestinal microbiome and metabolite profiling. Our results showed that both probiotics had a positive effect on growth, immunity, serum enzyme activity, the gut microbiome, and resistance to Aeromonas salmonicida in rainbow trout. Moreover, the intestinal microbial structure was reshaped with increased relative abundance of potential beneficial bacteria, such as *Ruminococcus*, *Lachnospiraceae ucg-004*, *Leptotrichia*, Bacillus coagulans, *Porphyromonadaceae*, *Anaerococcus*, and *Photobacterium* in the *B. velezensis* group and *Paenibacillaceae* and Eubacterium hallii in the *L. sakei* group. Metabolomic profiling and transcriptome analysis revealed upregulated metabolites as biomarkers, i.e., sucrose and l-malic acid in the *B. velezensis* group, and *N*-acetyl-l-phenylalanine, *N*-acetylneuraminic acid, and hydroxyproline in the *L. sakei* group. Additionally, a multiomics combined analysis illustrated significant positive correlations between the relative abundance of microflora, metabolites, and gene expression associated with immunity and growth. This study highlights the significant role of probiotics as effectors of intestinal microbial activity and shows that different probiotics can have a species-specific effect on the physiological regulation of the host. These findings contribute to a better understanding of the complex host-microbiome interactions in rainbow trout and may have implications for the use of probiotics in aquaculture.

**IMPORTANCE** Probiotics are kinds of beneficial live microbes that impart beneficial effects on the host. Recent studies have proven that when given supplementation with probiotics, farmed fish showed improved disease prevention and growth promotion. However, the underlying metabolic functions regarding their involvement in regulating growth phenotypes, nutrient utilization, and immune response are not yet well understood in the aquaculture field. Given the active interactions between the gut microbiota and fish immune and growth performance, we conducted the supplementation experiments with the probiotics Bacillus velezensis and Lactobacillus sakei. The results showed that probiotics mediated intestinal microbiome- and microbiota-derived metabolites regulating the growth and immunity of fish, and different probiotics participated in the species-specific physiological regulation of the host. This study contributed to a better understanding of the functional interactions associated with host health and gut microbiota species.

## INTRODUCTION

The increasing demand for high-quality protein has led to aquaculture being the fastest growing sector, representing an average consumption of 50% relative to total fish consumed per capita (1). Rainbow trout (Oncorhynchus mykiss) is one of the most commercially valuable cold-water species farmed worldwide (2). However, the intensification of aquaculture has led to long-term stress on farmed fish, making them more susceptible to pathogenic infections (3). This poses a major challenge for sustainable aquaculture of rainbow trout. Aeromonas salmonicida, in particular, is one of the most infective and lethal pathogens, causing serious furunculosis and huge economic losses (4). In addition, antibiotics, as the classic cure for microbial infection in the aquaculture industry, have been widely restricted due to the emergence of drug resistance genes (5, 6). Therefore, new strategies are required to address pathogen infection and the potential negative environmental effects associated with aquaculture.

Probiotics are live microbes that are beneficial to their host, and they have been shown to be effective in disease prevention and growth promotion in farmed fish (7, 8). Numerous studies have demonstrated the effects of different feed types containing probiotics, especially various strains from bacillus and lactobacillus, on growth and disease resilience (9–15). A promising probiotic strain of Lactococcus lactis (WFLU12) has shown strong inhibitory activity against bacterial pathogens and an increased specific growth rate in olive flounder (Paralichythys olivaceus) (16). Turbots (Scophthalmus maximus) supplemented with Bacillus coagulans exhibited better growth performance and increased digestion and absorption capacity (11). Probiotics can modulate both local mucosal and systemic immune responses, leading to reduced gut inflammation in many farmed fish (17). However, the underlying metabolic functions related to their involvement in the regulation of growth phenotypes, nutrient utilization, and immune response are not yet well understood, especially in aquaculture.

The intestinal microbiota plays an integral role in the development, physiology, and health of fish (18). Since the fish intestinal tract is a major site of pathogen transmission, manipulation of the gut microbiota using probiotics is a useful biological strategy to control pathogenic infections (19). However, most current studies only measure the effects of probiotics on overall fish growth or survival or simply describe the microbial composition of the gut environment, without conducting a comprehensive study to investigate complex host-microbiome interactions (20). In recent years, there has been increasing interest in revealing how intestinal bacteria interact with the host under the regulation of probiotics (21–23) or environmental influences (24, 25). In addition, the microbial end products are shown to act as signaling molecules regulating host immunity (26). In light of this, more in-depth studies are needed to identify these intermediate metabolites that serve as important immune promoters and to further understand the link between the observed altered metabolic phenotype and the fish intestinal microbiome.

In this study, rainbow trout were fed Bacillus velezensis MVCR2 and Lactobacillus sakei rMA-2 as dietary additives. A combined analysis of metabonomics, transcriptomics, and microbiomics was carried out to identify metabolites of the intestinal microbe manipulated by probiotic bacteria. The results of this study will contribute to a better understanding of the functional interactions associated with host health and gut microbiota species.

## RESULTS

### Fish growth, survival, and histological analysis.

**FIG 1:**
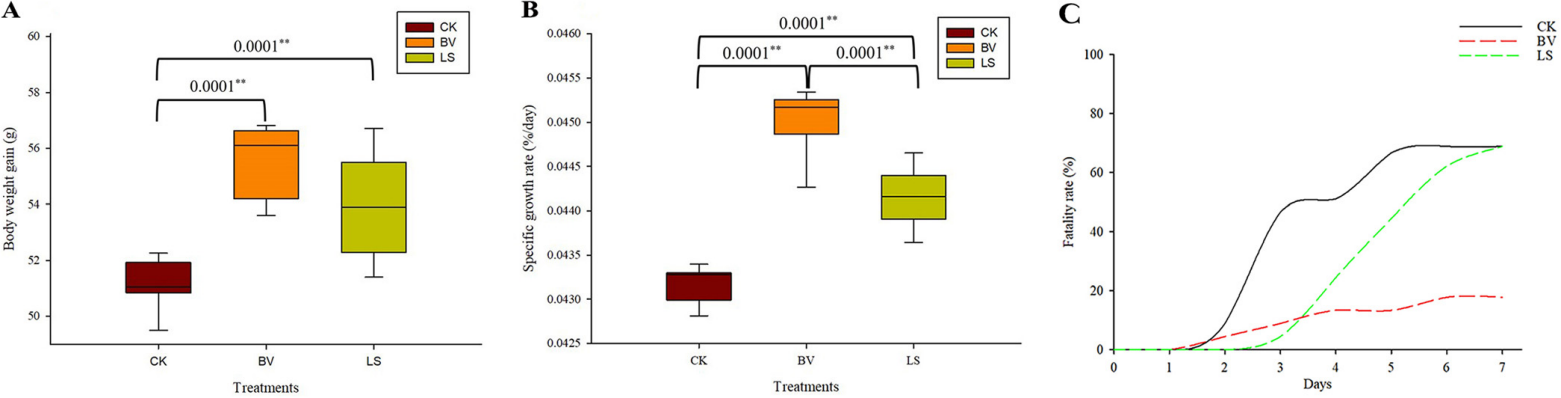
The effects of probiotics on the growth performance and fatality rate of rainbow trout. (A) Body weight; (B) specific growth rate; (C) fatality rate. CK, control group; BV, Bacillus velezensis MVCR2 strain supplementation group; LS, Lactobacillus sakei rMA-2 strain supplementation group. Data are given as the mean ± standard deviation (SD).

**FIG 2:**
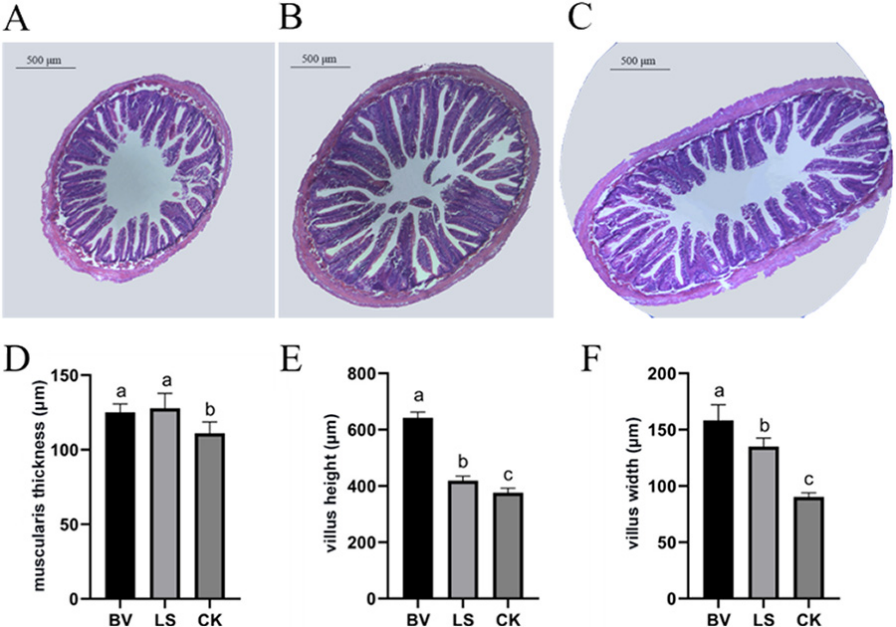
Morphology of the midintestine in rainbow trout after 30 days of feeding. (A) CK group; (B) Bacillus velezensis MVCR2 strain supplementation group; (C) Lactobacillus sakei rMA-2 strain supplementation group; (D) muscularis thickness; (E) villus height; (F) villus width. Data are given as the mean ± SD. Different letters above each bar indicate significant differences (*P < *0.05).

MVCR2 (*B. velezensis* group) and rMA-2 (*L. sakei* group) contributed to a higher body weight gain and specific growth rate (SGR) than the control (CK) group, with MVCR2 showing better results than rMA-2 (Fig. 1A and B). No fish mortality was observed before the *A. salmonicida* challenge, whereas after the challenge, fish mortality increased in all groups. The *B. velezensis* group had the highest survival rate after the challenge. Specifically, the *L. sakei* group had more surviving fish than the *B. velezensis* group the first during the 3 days of challenge. Unfortunately, the survival rate decreased to the level of the CK group on day 7 (Fig. 1C). Meanwhile, the histological results showed that the intestinal muscularis and villi of the treatment groups, especially in the *B. velezensis* group, increased in thickness and height (Fig. 2).

### Serum enzyme activities in the *B. velezensis*, *L. sakei*, and CK groups.

**FIG 3:**
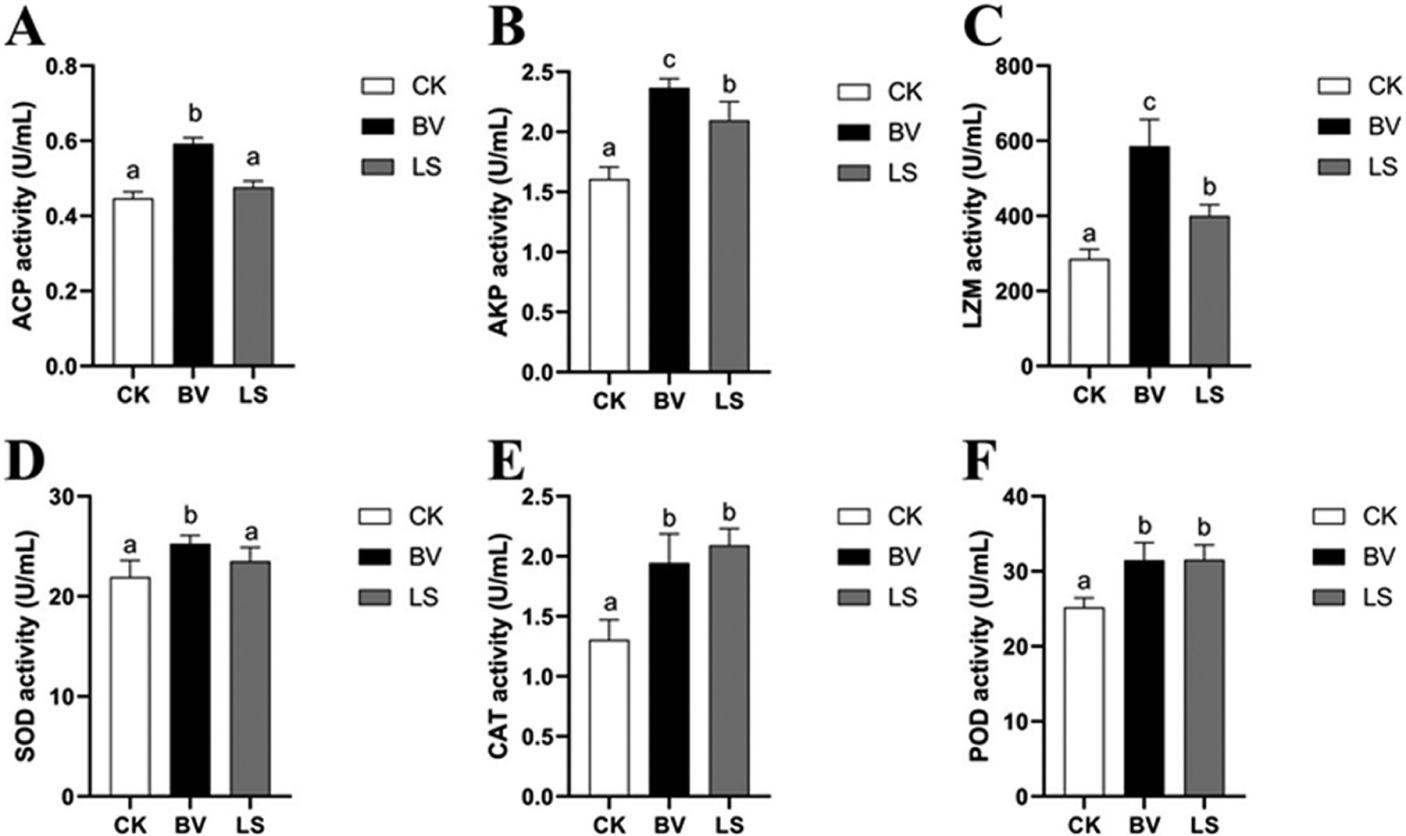
Serum immunity and antioxidant enzyme activities of rainbow trout. (A) Acid phosphatase activity; (B) alkaline phosphatase activity; (C) lysozyme activity; (D) superoxide dismutase activity; (E) catalase activity; (F) peroxidase activity. CK, control group; BV, Bacillus velezensis MVCR2 strain supplementation group; LS, Lactobacillus sakei rMA-2 strain supplementation group. Data are given as the mean ± SD. Different letters above each bar indicate significant differences among different groups (*P < *0.05).

The activities of enzymes (acid phosphatase [ACP], alkaline phosphatase [AKP], lysozyme [LZM], serum superoxide dismutase [SOD], catalase [CAT], and peroxidase [POD]) were significantly higher in the treatment groups than in the CK group (*P *< 0.05), except for ACP and SOD in the *L. sakei* group, which were significantly different than in the CK group (*P *> 0.05). In addition, the activities of enzymes (ACP, AKP, LZM, and SOD) in the *B. velezensis* group were significantly higher than those in the *L. sakei* group (*P *< 0.05) (Fig. 3).

### Intestinal transcriptome analysis.

**FIG 4:**
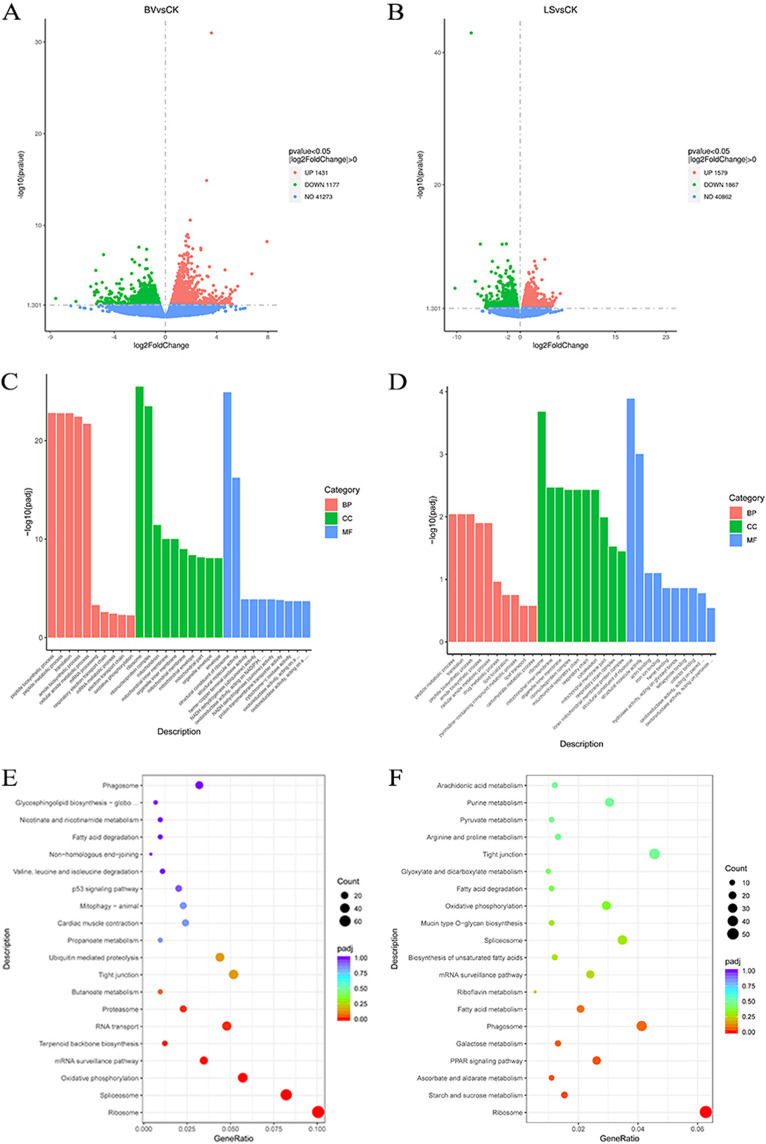
Intestinal transcriptome analysis. (A) Volcano map of the *B. velezensis* group. (B) Volcano map of the *L. sakei* group. (C) GO enrichment analysis of the *B. Velezensis* group. (D) GO enrichment analysis of the *L. sakei* group. (E) KEGG enrichment analysis of the *B. velezensis* group. (F) KEGG enrichment analysis of the *L. sakei* group. CK, control group; BV, Bacillus velezensis MVCR2 supplementation group; LS, Lactobacillus sakei rMA-2 supplementation group.

Transcriptome sequencing (RNA-Seq) results of the intestine transcriptome is shown in Table S2 in the supplemental material. The clean reads were mapped to the rainbow trout genome, with a mapped rate of 87.93% to 89.22%. The differential genes were selected based on |log_2_ (fold change)| > 0 and a *P* value of ≤0.05. The results indicate that probiotics have a considerable modulatory effect on intestinal transcriptomes. Notably, there is a difference between the *B. Velezensis* group and the *L. sakei* group in the presented transcripts. Specifically, a total of 1,431 upregulated genes and 1,177 downregulated genes were found in the *B. velezensis* group (Fig. 4A), and 1,579 upregulated genes and 1,867 downregulated genes were found in the *L. sakei* group (Fig. 4B). Further, Gene Ontology (GO) analysis of all the differentially expressed genes (DEGs) showed that biological processes (BPs), cell composition (CC), and molecular function (MF) were involved in the intestinal transcriptomes. The DEGs of the two treatment groups were mainly enriched in BPs that included peptide biosynthesis, peptide metabolism, translation, amide biosynthesis, and cellular amide metabolism; the CC processes included ribosomes and ribonucleoprotein complexes; and the MF processes included the structural composition and structural molecular activity of ribosomes. In addition, the *B. velezensis* group was enriched in mitochondria, while the *L. sakei* group was enriched in cytoskeleton according to CC analysis (Fig. 4C and D).

The analysis of KEGG pathways was performed to investigate the functional effects of the transcriptomic modulation with DEGs. According to KEGG analysis, the DEGs of the *B. velezensis* group were mainly enriched in eight metabolic pathways: ribosome (*n* = 76), spliceosome (*n* = 62), oxidative phosphorylation (*n* = 43), mRNA monitoring (*n* = 26), terpenoid main chain biosynthesis (*n* = 9), RNA transport (*n* = 36), proteasome (*n* = 17), and phagosome (*n* = 24) (Fig. 4E). On the other hand, the DEGs of the *L. sakei* group were mainly enriched in five metabolic pathways: including ribosome (*n* = 58), peroxisome proliferator-activated receptor (PPAR) signaling (*n* = 24), phagosome (*n* = 38), starch and sucrose metabolism (*n* = 14), and ascorbic acid and aldolic acid metabolism (*n* = 10) (Fig. 4F).

Several DEGs related to immunity and growth were validated using quantitative PCR (qPCR) analysis (Fig. S1 and S2). The qPCR results were consistent with the transcriptome data. In the *B. velezensis* group, immune-related genes (*lectin*, *lyg*, *hsp70b*, *il-16*, and *tradd*), a growth regulation gene (*igfbp2*) and pathway-related genes *rpl35a* (ribosomal pathway) and *sem1* (proteasome pathway) were all upregulated. In the *L. sakei* group, immune-related genes (*siglec11*, *mrc2*, *adam17*, and *itga5*), a growth-related gene (*igfbp2*) and pathway-related genes *rpl35a* and *pygmb* (starch and sucrose metabolism pathway) were also upregulated.

### Gut microbiota structure altered by probiotic supplementation.

**FIG 5:**
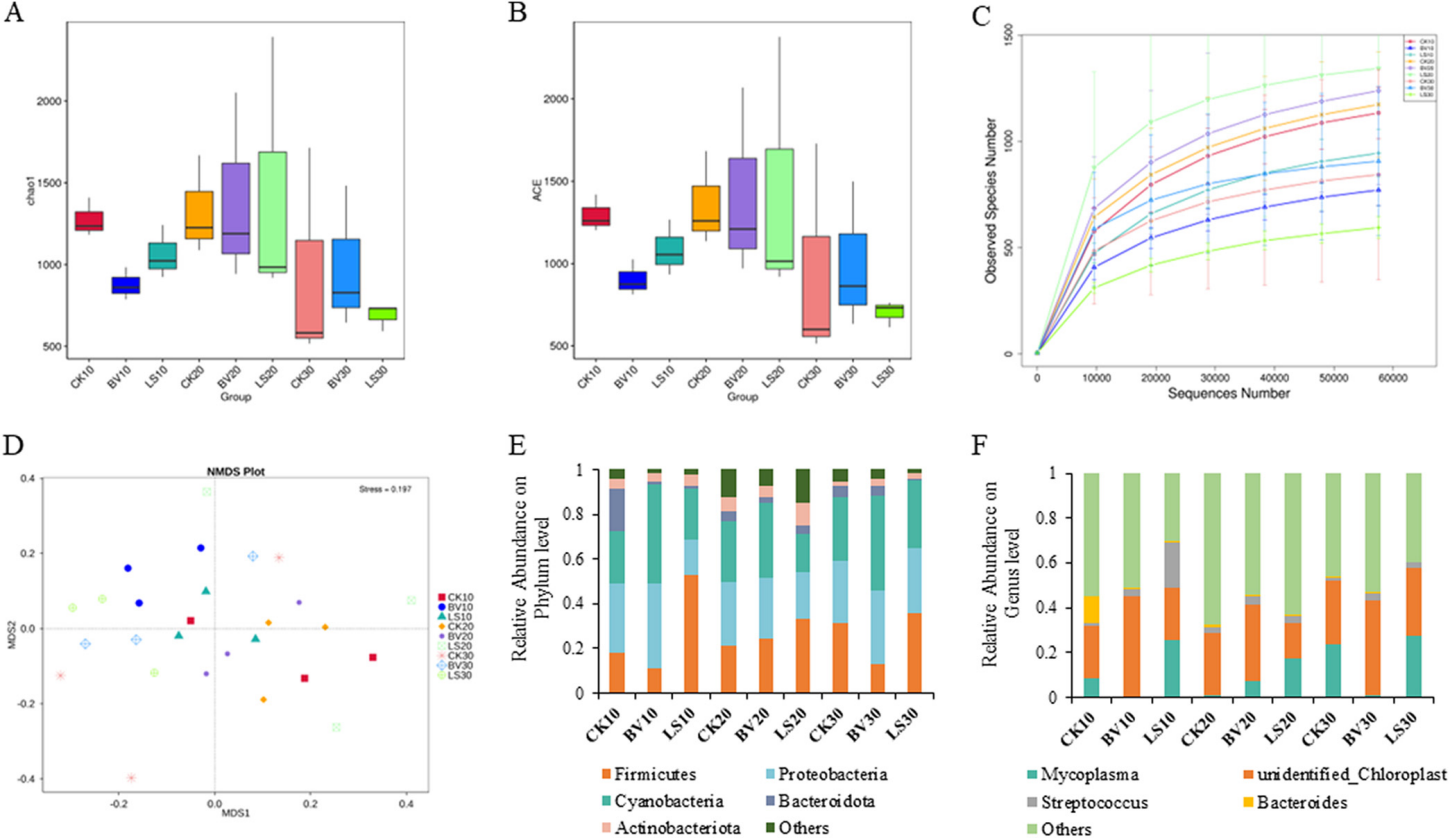
Diversity and taxonomic classification of intestinal flora in rainbow trout. (A) Chao1; (B) ACE; (C) rarefaction curves based on the sequencing data; (D) NMDS analysis based on Bray-Curtis distance; (E) phylum level classification of intestinal flora; (F) genus level classification of intestinal flora. CK, control group; BV, Bacillus velezensis MVCR2 supplementation group; LS, Lactobacillus sakei rMA-2 supplementation group.

The diversity and richness of the intestinal microbiota were calculated in the treatment groups and the CK group. A total of 5,618 operational taxonomic units (OTUs) were obtained with 97% identity, and 2,480 (44.14%) were annotated to the genus level. The Chao1 index and abundance-based coverage estimator (ACE) index showed a significant increase in the *B. velezensis* group compared to the CK group after 30 days of feeding (Fig. 5A and B). The rarefaction curves indicated that the sequencing depth was sufficient to describe the bacterial diversity of the samples (Fig. 5C). As shown by nonmetric multidimensional scaling (NMDS) analysis, the *B. velezensis* and *L. sakei* groups had a remodeled structure of gut microbiota (Fig. 5D).

The five most abundant microbiota found in the intestine were identified at the phylum and genus levels. The five most abundant phyla were *Firmicutes*, *Proteobacteria*, *Cyanobacteria*, *Bacteroidota*, and *Actinobacteria*, accounting for 87.6% to 98.7% of the total gut microbiota. In the *B. velezensis* group, the relative abundances of *Proteobacteria* and *Cyanobacteria* increased, while the relative abundance of *Firmicutes* decreased at day 10 and day 30. In the *L. sakei* group, the relative abundances of *Proteobacteria* and *Bacteroidota* decreased, while *Firmicutes* increased its abundance during the experiment (Fig. 5E). At the genus level, the relative abundance of *Mycoplasma* and unidentified *Chloroplast* was the highest, accounting for 37.36% (CK group), 43.20% (*B. velezensis* group), and 46.62% (*L. sakei* group) of the total gut microbiota. Notably, the highest abundance of unidentified *Chloroplast* was found in the *B. velezensis* group, while the relative abundance of *Mitochondria* accounted for a lower proportion than in the CK and *L. sakei* groups (Fig. 5F).

Differences in abundant taxa were determined using linear discriminant analysis effect size (LEfSe) analysis with linear discriminant analysis (LDA) >2.5 (*P *< 0.05). In the *B. velezensis* group, some potential pathogenic bacteria and environmental super-multidrug-resistant bacteria, such as *Mycoplasma*, *Ralstonia*, *Pedobacter*, Staphylococcus, *Neisseria*, and *Xanthomonas*, decreased their abundances; the genera containing more beneficial bacteria, including *Ruminococcus*, *Lachnospiraceae ucg-004*, *Leptotrichia*, Bacillus coagulans, *Porphyromonadaceae*, *Anaerococcus*, and *Photobacterium*, increased their abundances (Fig. S3A to C). In the *L. sakei* group, the genera involving more potentially pathogenic bacteria decreased their abundances, such as *Burkholderiales*, Staphylococcus, *Chryseobacterium*, *Erysipelotrichaeles*, *Peptostreptococcaceae*, and Escherichia coli; the genera containing more beneficial bacteria were increased, such as *Paenibacillaceae* and Eubacterium hallii (Fig. S3D to F).

### Fecal metabolomics alterations in the probiotic supplementation and control groups.

A comprehensive metabolic analysis of the intestinal contents obtained in our experiment was performed. The partial least-squares discriminant analysis (PLS-DA) score plot showed significant changes in the metabolic phenotype of the treatment groups (Fig. S4A and C). The model parameters of R2Y and Q2Y, along with the validation plot of the PLS-DA model, confirmed the reliability of the model (Fig. S4B and D).

**FIG 6:**
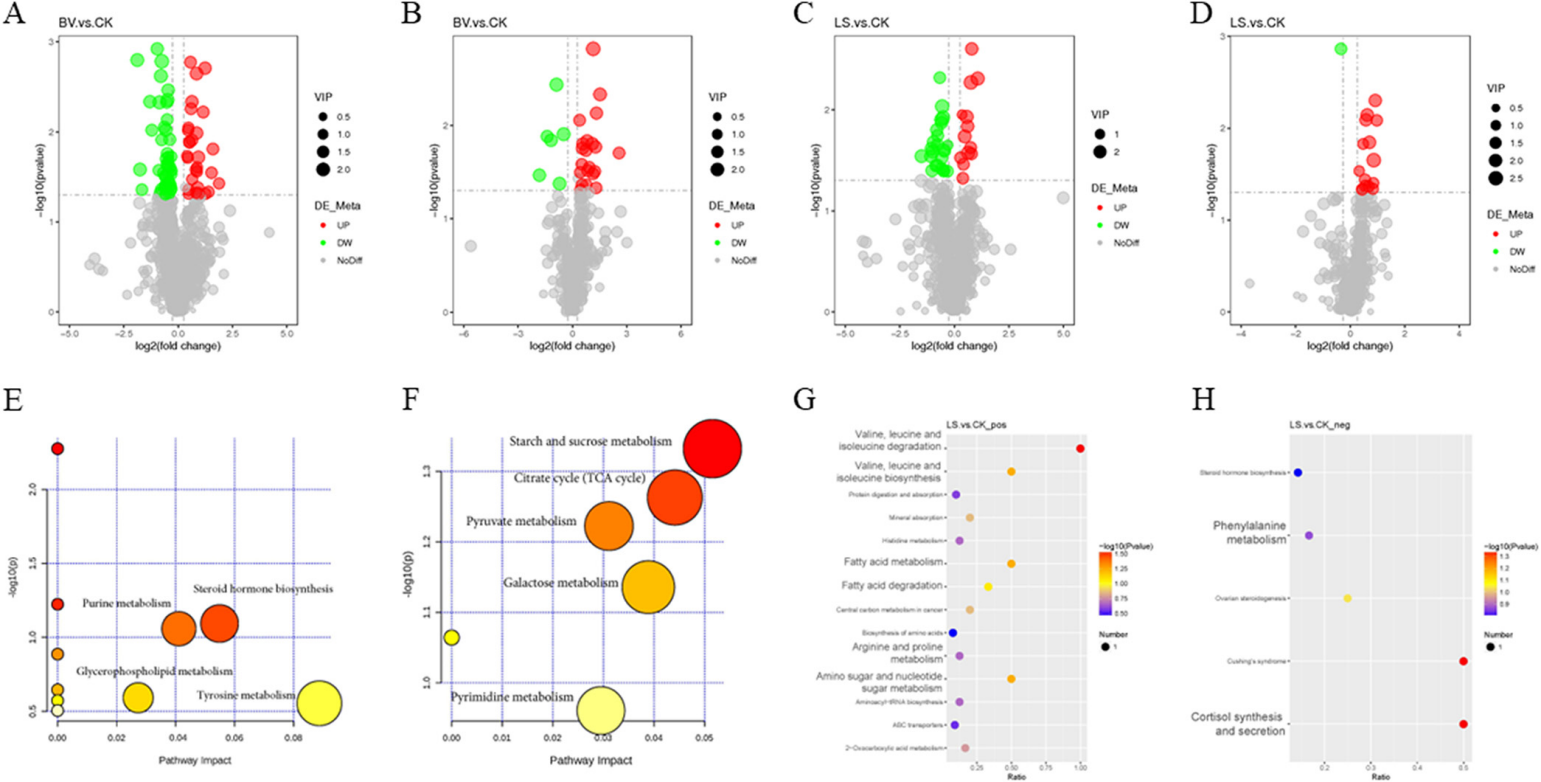
The differential metabolites in intestinal metabolomics. (A to D) Volcano map of differential metabolites. (A and C) Positive ionization mode; (B and D) negative ionization mode. The horizontal coordinate represents the multiple change of difference (log_2_ fold change) of metabolites in different groups, and the vertical coordinate represents the significance level of difference (–log_10_ *P* value). Each point in the volcano map represents a metabolite, and the significantly upregulated metabolites are represented by red dots, while the significantly downregulated metabolites are represented by green dots. The dot size represents the VIP value. (E to H) KEGG enrichment pathway; (E and G) positive ionization mode; (F and H) negative ionization mode.

A total of 104 differential metabolites were identified in the *B. velezensis* group, with 50 being upregulated and 54 being downregulated (Fig. 6A and B). A total of 53 differential metabolites were identified in the *L. sakei* group, with 28 being upregulated and 25 being downregulated (Fig. 6C and D). The identified differential metabolites were classified and annotated using the KEGG database to determine the main biochemical metabolic pathways. The KEGG annotation analysis showed that the most enriched biochemical metabolic pathways in the *B. velezensis* group were starch and sucrose metabolism, pyruvate metabolism, carbohydrate digestion and absorption, citric acid cycle (tricarboxylic acid [TCA] cycle), glyoxylic acid and dicarboxylic acid metabolism, galactose metabolism, ABC transporter, gap junction, salivary secretion β-alanine metabolism, vascular smooth muscle contraction, etc. (Fig. 6E and F). The corresponding upregulated metabolites, starch, sucrose, and l-malic acid, identified in the *B. velezensis* group were enriched in the most important metabolic pathways. The most enriched pathways in the *L. sakei* group were amino sugar and nucleotide sugar metabolism, arginine and proline metabolism, phenylalanine metabolism, biosynthesis and degradation of valine, leucine and isoleucine, fatty acid metabolism and degradation, cortisol synthesis and secretion, Cushing’s syndrome, etc. (Fig. 6G and H). Among the corresponding metabolites in enriched pathways of the *L. sakei* group, *N*-acetyl-l-phenylalanine, *N*-acetylneuraminic acid, and hydroxyproline were identified as the most significantly upregulated.

### Multiomics combined analysis and correlation of gut microbiota, metabolites, and DEGs.

**FIG 7:**
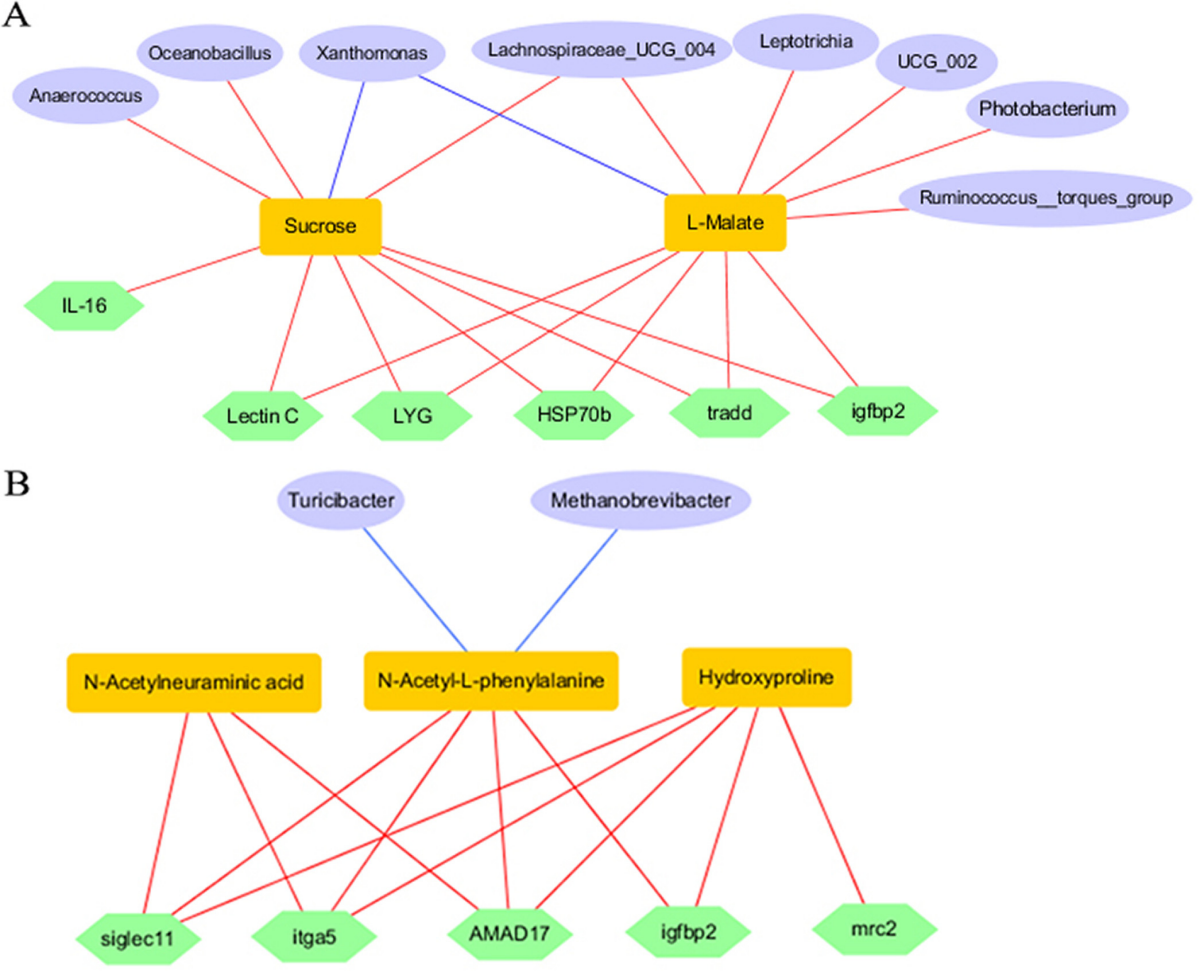
Combined analysis of microflora, metabolites, and expression of genes. (A) The correlation in the *B. velezensis* group. (B) The correlation in the *L. sakei* group. The red and blue lines show the significant positive and negative correlations, respectively, among microflora, metabolites, and expression of genes (*P* < 0.05).

The relationship between gut microbiota, metabolites, and DEGs was evaluated using Pearson correlation analysis. In the *B. velezensis* group, the relative abundance of *Anaerococcus*, *Lachnospiraceae UCG-004*, and *Oceanobacillus* was significantly positively correlated with the production of sucrose (*P < *0.05); *Photobacterium*, *Leptotrichia*, *Lachnospiraceae UCG-004*, and *Ruminococcus* were significantly positively correlated with the production of l-malate (*P *< 0.05). Meanwhile, the production of sucrose and l-malate was positively correlated with the DEGs, such as *lectin*, *lyg*, *hsp70b*, *il-16*, *tradd*, and *igfbp2* (Fig. 7A). In the *L. sakei* group, *Methanobrevibacter* and *Turicibacter* were negatively correlated with the production of *N*-acetyl-l-phenylalanine (*P *< 0.05). Meanwhile, the production of *N*-acetyl-l-phenylalanine, *N*-acetylneuraminic acid, and hydroxyproline were significantly positively correlated with DEGs, including *siglec11*, *mrc2*, *adam17*, *itga5*, and *igfbp2* (Fig. 7B).

**FIG 8:**
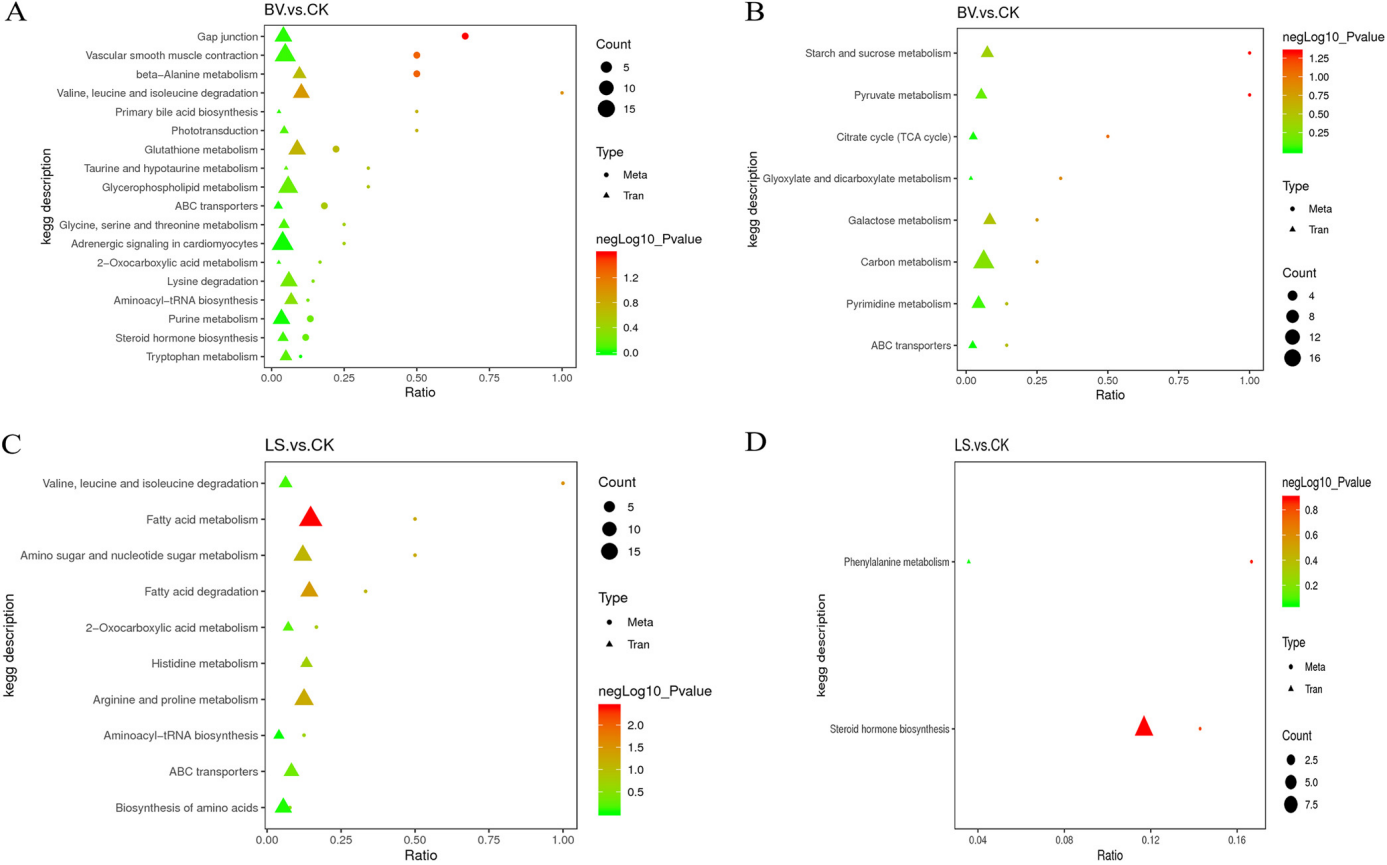
Combined analysis of transcriptomes and metabolomes. (A) The positive ionization mode in the *B. velezensis* group. (B) The negative ionization mode in the *B. velezensis* group. (C) The positive ionization mode in the *L. sakei* group. (D) The negative ionization mode in the *L. sakei* group. The triangles represent the DEGs in the transcriptome. The circles represent the differential metabolites in the metabolome.

A combined analysis of transcriptomes and metabolomes was conducted to map the DEGs and metabolites to the KEGG pathway database in order to determine the main biochemical pathways and signal transduction pathways in which the differential metabolites and DEGs participate. In the *B. velezensis* group, pathways enriched in starch and sucrose metabolism, pyruvate metabolism, the tricarboxylic acid cycle, glyoxylic acid and dicarboxylic acid metabolism, galactose metabolism, ABC transporter, etc. were found (Fig. 8A and B). In the *L. sakei* group, pathways enriched in arginine and proline metabolism, amino sugar and nucleotide sugar metabolism, phenylalanine metabolism, valine, leucine, and isoleucine degradation, fatty acid metabolism, fatty acid degradation, amino acid biosynthesis, histidine metabolism, etc. were found (Fig. 8C and D).

## DISCUSSION

Most current studies in the field of aquaculture have largely focused on verifying the positive impacts of various probiotic strains on fish production and disease prevention (27–31). However, the underlying mechanism of these effects remain unknown. Our results confirmed that the use of probiotic strains (MVCR2 and rMA-2) had a positive impact on the growth, physiology, and intestinal flora of rainbow trout, even in the presence of *A. salmonicida* infection. We observed morphological changes in the intestinal muscularis (32, 33) and an improvement in the serum immune index (ACP, AKP, LZM) (13–15, 34–37), which likely contributed to the positive effects. Notably, MVCR2 had a greater effect than rMA-2, indicating that the metabolic regulatory effects of different probiotic strains may be species-specific. Recent advances have shown that the gut microbiota is involved in the metabolic homeostasis and energy balance of the host. Therefore, a comprehensive understanding of the networks of host-microbiome interactions that integrate multiomics data is expected to further advance in the field.

Probiotics supplementation has been shown to activate the innate immune response in the fish host (38). For example, in rainbow trout, dietary supplementation of the probiotic bacterium Lactobacillus rhamnosus triggered the expression of immune-related genes, such as interleukin-1 (*il-1*) and tumor necrosis factor-alpha (*TNF-1α*), which subsequently led to a higher survival rate after Yersinia ruckeri challenge (39); feeding the probiotic Saccharomyces cerevisiae resulted in the upregulation of lectin in rainbow trout (40). Transcriptomic analysis has shown that probiotic-fed fish exhibit a shift in functional effects in signaling pathways, immune-related pathways, protein digestion and absorption, and starch and sucrose metabolism (40). In this study, transcriptome and qPCR analyses showed that a series of immune-related genes was upregulated after supplementation of dietary MVCR2 and rMA-2, and these results were consistent with changes in serum physiological indices. The activation of immune-related genes in this study supports the fact that dietary MVCR2 and rMA-2 improved the pathogen resistance to *A. salmonicida* in rainbow trout. Additionally, we observed upregulation of the growth regulation-related gene *igfbp2* in both treatment groups. *igfbp2*, a member of the IGFBP family, plays a key role in regulating cell proliferation and metabolism to control development and growth. Based on KEGG analysis of the transcriptome data, it appears that supplementation of MVCR2 has a regulatory effect on genetic information processing, immune-related pathways, and oxidative phosphorylation. On the other hand, supplementation with rMA-2 mainly regulates ribosomes, phagosomes, fatty acid metabolism, the PPAR signaling pathway, and the metabolic pathway. These results indicated that the two probiotic strains act on different metabolic pathways and play distinct roles in boosting host metabolism. Several studies have shown that symbiotic microbes can activate proinflammatory genes via the TLR/MyD88 signaling pathway, which can enhance disease resistance in fish (41). However, in this study, based on transcriptome data from day 30, it appears that the intestinal TLRs were not activated. Our previous study suggested that during the early stages of probiotic use, different Toll-like receptor (TLR)-mediated MyD88-independent pathways may be activated in the liver and spleen, but TLRs were not always upregulated through the use of probiotics. Nonetheless, TLRs seem to be trained at an early stage to respond more rapidly in the face of pathogen invasion (38). Therefore, different strains of probiotics with species specificity exert their probiotic functions by regulating different metabolic pathways in fish at different time periods.

Intestinal bacterial communities have been increasingly recognized as playing an important role in shaping the health of animals. Probiotics alter the intestinal flora and interact with them to produce different types of metabolites or antimicrobial agents that affect host health. Therefore, target manipulation of the gut microbiota has been shown to have protective effects against pathogen infections (42). Based on microbiome analysis, dietary MVCR2 and rMA-2 induced changes in the structure and abundance of the gut microbiota in different ways, which potentially affected the growth and immunity of fish. Specifically, MVCR2 supplementation caused a significant increase in the community richness and diversity of intestinal flora. In particular, the abundances of core intestinal flora, including *Firmicutes*, *Proteobacteria*, *Cyanobacteria*, *Bacteroidota*, and *Actinobacteriota* were changed. *Firmicutes* is reported to participate in lipid metabolism in which lactic acid bacteria can promote its abundance and dominance in the intestine (43). Notably, in this study, the abundance of *Firmicutes* decreased after MVCR2 supplementation and increased after rMA-2 supplementation. *Proteobacteria* is the dominant phylum in the gut microbiota of many marine and freshwater fishes and is involved in the degradation of carbon complexes and nitrogen (44). In this study, MVCR2 supplementation resulted in a decrease in the abundance of *Proteobacteria*. *Cyanobacteria* was shown to provide a promising source for producing novel natural products with potent biological activities (45), and this taxon was found to be more abundant in the MVCR2-fed group. In addition, the *Firmicutes*/*Bacteroidota* (F/B) ratio of the gut microbiota is recognized as an eventual biomarker related to weight gain and maintenance of intestinal homeostasis in humans (46, 47). In this study, the abundance of *Firmicutes* and *Bacteroidota* changed in probiotic-fed fish, suggesting a potential relationship to weight gain. Obviously, more studies are required to evaluate the ratio of F/B or other microbiota to fish health.

The probiotic-oriented regulation of the gut microbiota is based on understanding the composition of intestinal flora and the corresponding phenotype (48). In this study, we demonstrate that supplementation with MVCR2 and rMA-2 increased the abundance of beneficial microbes while decreasing the abundance of harmful microbes. Specifically, a high abundance of *Ruminococcus*, *Lachnospiraceae UCG-004*, and *Anaerococcus* contributed to butyrate production (49), while *Porphyromonadaceae* was capable of producing short-chain fatty acids (SCFAs) (50). Additionally, *Paenibacillus* was found to produce extracellular polysaccharides and enzymes (51), Eubacterium hallii produced acetate, butyrate, propionate, and formate (52), and *Oceanobacillus* is known for its potential to produce various enzymes, antibiotics, and exopolysaccharides (53). The production of these metabolites is consistent with the results of the metabolome in this study. The increase in the abundance of beneficial microbes may have the potential to improve intestinal function and nutrient processing ability, which could be reflected in growth and intestinal histomorphology.

There is growing evidence that the intestinal microbiota may regulate host growth by altering its own metabolic capacity (34, 48, 49, 54). Bacterial metabolites have been shown to provide an important link between the intestinal flora and physiological homeostasis of the host (55). Our findings suggest that MVCR2 mediates carbohydrate metabolism, promoting the glycolytic pathway and TCA cycle to regulate starch and sucrose metabolism. Sucrose and l-malic acid metabolites were enriched as intermediates in the glycolytic pathway and tricarboxylic acid cycle (TCA cycle). Previous studies of brook trout (Salvelinus fontinalis) and sea bream (Sparus aurata) have shown that dietary starch is better utilized as an energy source than dietary glucose (56, 57). Meanwhile, malic acid is an essential intermediate in the TCA cycle, and upregulation of the TCA cycle further promoted the utilization of sucrose. In addition, an activated TCA cycle and elevated malic acid have also been found to enhance the survival of zebrafish against Vibrio alginolyticus (58). Based on the conjoint analysis, we also found that increased abundance of beneficial microbes such as *Anaerococcus*, *Lachnospiraceae UCG-004*, and *Oceanobacillus* was associated with sucrose production, whereas *Photobacterium*, *Leptotrichia*, *Lachnospiraceae UCG-004*, and *Ruminococcus* were involved in l-malate production. Therefore, we speculated that MVCR2 supplementation increased the abundance of beneficial intestinal microbes to produce sucrose and l-malic acid metabolism and contribute to the glycolytic pathway and TCA cycle. In this study, rMA-2 supplementation may have contributed to the amino acid biosynthesis pathway to affect host gut health by increasing the production of hydroxyproline, *N*-acetylneuraminic acid and *N*-acetyl-l-phenylalanine. Hydroxyproline is an important amino acid with specific physiological or biochemical functions that may be metabolically restricted. Supplementation of hydroxyproline has been shown to improve growth and development in salmonids, such as Salmo salar L (59). The presence of *N*-acetylneuraminic acid may have an effect on binding of *A. salmonicida* in the intestine and plays a role in the mucosal defense of Atlantic salmon (60). l-phenylalanine is an important essential amino acid that is metabolized by the microbiota into a large group of downstream proteins that can be absorbed by the gut and incorporated into host proteins (61). The rMA-2-induced production of hydroxyproline, *N*-acetylneuraminic acid and *N*-acetyl-l-phenylalanine is associated with the DEGs (e.g., *siglec11*, *mrc2*, *ADAM17*, *itga5*, and *igfbp2*) which were found to be positively corrected. *Methanobrevibacter* and *Turicibacter* were negatively correlated with the production of *N*-acetyl-l-phenylalanine. Furthermore, the results of predicted metabolic pathways confirmed a regulatory effect of the production of key metabolites in the intestinal flora on host growth and immunity. Obviously, more studies are required to verify the functions and mechanism of the identified single or mixed metabolites in the regulation of growth and immunity in fish, as well as the feedback effect of the intestinal microbiota.

This study revealed that the use of probiotic strains can mediate the changes in the gut microbiota and metabolite production that trigger growth and immune-related pathways in rainbow trout. Specifically, MVCR2 supplementation increased the abundance of beneficial gut microbes to produce sucrose and l-malic acid, promoting the glycolytic pathway and the TCA cycle. The supplementation of rMA-2 may contribute to the amino acid biosynthesis pathway, which ultimately affects host gut health by increasing the production of hydroxyproline, *N*-acetylneuraminic acid, and *N*-acetyl-l-phenylalanine. The present study provides molecular evidence for host-gut microbiome interactions using multiomics data. In addition, the effects of probiotics on host health are species specific.

## MATERIALS AND METHODS

### Fish culture and experiment design.

The average weight of rainbow trout was 19.4 ± 0.5 g. Rainbow trout were obtained from a hatchery farm located in Linqu Shandong Province, China, and transported to the laboratory of Comprehensive Aquaculture Center, School of Marine Science and Engineering, Qingdao Agricultural University using plastic bags filled with pure oxygen. The fish were cultured in a flow-through water system consisting of 12 tanks, each with a volume of 300 L (diameter, 97 cm; height, 87 cm). After 2 weeks of acclimation, a 30-day feeding experiment was conducted. Three groups of fish were included in this study and were fed different diets: dietary supplementation of Bacillus velezensis strain MVCR2 (*B. velezensis* group), Lactobacillus sakei strain rMA-2 (*L. sakei* group), and a control group without any probiotic supplementation (CK group). Each group had four repetitions, with 50 individual fish per tank. The tested probiotics were coated on the commercial diets. The fish were fed twice per day (08:00 and 17:00) at a rate of 2.0% of their body weight. The water in the tank was exchanged at a rate of 50% daily. The water temperature was maintained at 14.5 ± 0.5°C, and the pH was maintained at 7.0 ± 0.3. The dissolved oxygen (DO) was above 6.5 mg/L, and the photoperiod was set at 14 h (light):10 h (dark). All experimental procedures were conducted strictly in accordance with the research protocols approved by Qingdao Agricultural University for the ethical treatment of experimental animals.

### Probiotics strains.

The strains used in this experiment were Bacillus velezensis MVCR2 and Lactobacillus sakei rMA-2, which were isolated from the intestines of rainbow trout and turbot. The bacterial solutions of strains MVCR2 and rMA-2 were cultured to a concentration of 1.0 × 10^9^ CFU/mL using Luria-Bertani broth and de Man-Rogosa-Sharpe broth culture medium, respectively. The *in vivo* safety and characterization and the *in vitro* antibacterial effects on *A. salmonicida* of the two strains were checked before use.

### Diets and culture conditions.

The parameters of the probiotic strains, diets, and culture conditions, as well as calculation of growth performance and survival rates, were well described in our previous study (38). The commercial diets used in this experiment were provided by Qingdao Aile Co. Ltd. and were designed for rainbow trout. The main components of the basal diet included 45.0% crude protein, 20.0% crude lipid, 3.0% crude fiber, 9.0% crude ash, 2.0% total phosphorus, 0.8% calcium, and 3.3% amino acids. The diets were sterilized using thermal disinfection at 100°C prior to use. The probiotics were diluted with distilled water and coated on the surface of the sterilized diet. The dose of living bacterial strains was calculated as 1.0 × 10^7^ CFU/g using optical density (OD) values.

After the feeding experiment, rainbow trout were injected intraperitoneally with 100 μL of *A. salmonicida* at a concentration of 5 × 10^7^ CFU/mL. The cumulative mortality of the rainbow trout was observed and calculated over the next 7 days.

### Sampling.

Six individual fish were sampled from each tank, and blood samples and intestine and gut contents were collected on day 10, day 20, and day 30. Prior to sampling, the fish were euthanized with MS-222 (ethyl 3-aminobenzoate methanesulfonate). The intestine and gut contents were packed into separate sterile tubes and put in liquid nitrogen immediately. The blood was drawn into 1.5-mL centrifuge tubes and stratified and centrifuged at 3,000 rpm for 20 min. The supernatant was drawn into a new centrifuge tube and then stored at −80°C before analysis.

### Growth, survival, and histological analysis.

At the end of the feeding experiment, food was withheld from the fish 24 h, and the fish were weighed using a balance. The weight gain (WGR), specific growth rate (SGR), and fatality rate (FR) were calculated as follows:

WGR (%) = 100% × (BWF – BWI)/BWI

SGR (% d−1) = 100% × (ln BWF – ln BWI)/Df

SR (%) = 100%× Nd/Ni

where BWI, BWF, Df, Ni, and Nf represent the initial body weight, finial body weight, the duration of the experiment in days, the initial fish numbers, and the dead fish numbers, respectively.

Midintestine samples of day 30 were used for the histological assay. Samples were fixed in 4% paraformaldehyde solution for 24 h, dehydrated in ethanol, equilibrated in xylene, and then embedded in paraffin. Paraffin blocks were sectioned (7 μm) using a Leica RM 2135 rotary sectioner, stained with hematoxylin and eosin (HE), observed under a Zeiss Axio scope, and photographed with a Zeiss Axiocam 305 color microscope camera.

### Activities of serum enzymes.

Serum enzyme activities, i.e., acid phosphatase (ACP), alkaline phosphatase (AKP), lysozyme (LZM), catalase (CAT), serum superoxide dismutase (SOD), and peroxidase (POD) were measured using kits (Nanjing Jiancheng Bioengineering Institute, China) according to the manufacturer’s instructions.

### Transcriptome analysis.

Total RNA was extracted using TRIzol reagent (Agilent Technologies, USA). RNA quality was analyzed using agarose gel electrophoresis and Agilent 2100 Bioanalyzer (Agilent Technologies). The mRNA was enriched from total RNA using magnetic beads with oligo(dT) and was randomly broken into small pieces in fragmentation buffer. The first-strand cDNA was synthesized, and the 370- to 420-bp cDNA was screened using AMPure XP beads. The final cDNA library was acquired after the intact cDNA amplification and purification. The samples were pooled for sequencing using the Illumina platform. The high-quality clean data were mapped to the reference genome using HISAT2 v2.0.5. The mapped sequence coverage was counted using Feature Counts v1.5.0-P3. The reads per kilobase per million (RPKM) metric was calculated by normalizing the sequence coverage over the gene length. The differential expression was analyzed using DESeq2 R v1.20.0. GO and KEGG analysis of all differentially expressed genes was performed using clusterProfiler v3.4.4.

### Real-time quantitative PCR (qPCR) analysis.

The expression levels of 14 genes related to immunity and growth were verified using qPCR analysis. Primers were designed using Primer Premier 3 and are shown in Table S1. *EF1-α* and β-*actin* were used as reference genes. The methods for qPCR were similar to those in our previous study (11). Total RNA was extracted using RNAiso Plus (TaKaRa, Japan). The quality and concentration of RNA were analyzed using 1.0% agarose gels electrophoresis and a spectrophotometer (ND-2000, Nanodrop, USA). The cDNA was synthesized with a PrimeScript RT reagent kit with gDNA Eraser (Perfect Real Time, TaKaRa) and diluted to a final concentration of 50 ng/μL with sterilized double-distilled water. qPCR was performed with TB Green premix *Ex Taq* II (TaKaRa) in a LightCycler 96 real-time PCR system (Roche, Basel, Switzerland) with reactions of 10-μL volumes. Each assay was carried out in triplicate. Data analysis was conducted using the 2^–ΔΔ^*^CT^* method.

### Intestinal microbiome analysis.

Genomic DNA was extracted from the content of intestine samples using the cetyltrimethylammonium bromide (CTAB) method. The purity and concentration of the obtained genomic DNA were determined using a NanoDrop 2000 device (Thermo Scientific, Wilmington, NC, USA). The quality of the genomic DNA was checked by 1% agarose gel electrophoresis. The V3 to V4 region of the 16S rRNA gene was amplified by PCR using primers 341F (5′-CCTAYGGGRBGCASCAG-3′) and 806R (3′-GGACTACNNGGGTATCTAAT-5′). The purified amplicons were pooled using a TruSeq DNA PCR-free sample preparation kit (Beckman Coulter Life Sciences, USA) and sequenced on NovaSeq 6000 platform.

Raw sequencing data were merged using FLASH software (v1.2.7). High-quality sequencing data were filtered using Trimmomatic (v0.33) according to overlapping bases. QIIME software (v1.9.1) through the website https://github.com/torognes/vsearch/ was used to remove chimeras to acquire the qualified data.

Operational taxonomic units (OTUs) were clustered using qualified data according to 97% similarity using UPARSE (v7.0.1001). Taxonomic classifications for each OTU were analyzed using Mothur software with a 0.80 confidence threshold. The phylogenetic investigation of OTUs was constructed with MUSCLE (v3.8.31). Alpha diversity, including Chao1, Shannon, Simpson, ACE, and Good’s coverage, was calculated using QIIME software (v1.9.1). The principal-coordinate analysis (PCoA) plots were evaluated using R software (v2.15.3) based on weighted correlation network analysis (WGCNA), statistics, and the ggplot2 package. Linear discriminant analysis (LDA) effect size (LEfSe) analysis was performed to identify the differential bacterial taxa between groups.

### Metabonomics analysis.

The intestinal contents were homogenized using liquid nitrogen. Homogenized samples were mixed with 500 μL of methanol (80%) in a new Eppendorf tube in an ice bath for 5 min and centrifuged at 15,000 × *g* for 20 min at 4.0°C to obtain the supernatant. The supernatant was diluted with mass spectrometry water to a methanol concentration of 53%. The sample was centrifuged at 15,000 × *g* for 20 min at 4.0°C, and the resulting supernatant was used for liquid chromatography-mass spectrometry (LC-MS) analysis. Six biological replicates were used for each group. A quality control (QC) sample was prepared by mixing equal aliquots of the supernatants from each sample with the black sample using 53% methanol.

The LC-MS analyses were performed using Vanquish ultra-high-performance liquid chromatography (UHPLC; Thermo Fisher, Germany) in a Q Exactive HF spectrometer (Thermo Fisher, Germany) coupled with a Hypersil Gold column (Thermo Fisher, USA) in both electrospray ionization (ESI) positive and ESI negative ion modes. The flow rate was 0.2 mL/min, and the column temperature was 40°C. The binary gradient elution system included a positive mode containing 0.1% formic acid (A) and methanol (B) and a negative mode containing 5 mM ammonium acetate (pH 9.0) (A) and methanol (B). The gradient separation conditions were the following: 0 min, 1.5 min, 98% A and 2% B; 12 min, 14 min, 0% A and 100% B; 14.1 min, 17 min 4 min, 98% A and 2% B. The full scan *m/z* ranges were from 100 to 1,500. The parameters of mass spectrometry were as follows: spray voltage was kept at 3.2 kV, sheath gas flow rate was 40 arb, aux gas flow rate was 10 arb, and capillary temperature was 320°C. The MS/MS was done with data-dependent scans.

The raw data were analyzed and processed using Compound Discoverer v3.1 software (CD3.1, Thermo Fisher) for peak detection, extraction, alignment, and integration. The quantitative results were obtained by alignment of the prediction of the ion peak using the mzCloud (https://www.mzcloud.org/), mzVault, and Masslist databases. Background ions were removed with blank samples. For statistical analysis, partial least-squares discriminant analysis (PLS-DA) was used to analyze the significant metabolites between the control and treatment groups using the R package and build the PLS-DA model. The models were validated through 200 times of permutation tests. The model validity was verified by a 200-permutation test. The differential metabolites were screened on the basis of the combination of a statistically significant threshold of variable importance in the projection (VIP) value and fold change (FC) obtained from the PLS-DA model and *P* values from a two-tailed Student’s *t* test. The metabolites with VIP values of >1.0, FC of >1.2 or FC of <0.833, and a *P* value of <0.05 were considered differential metabolites. The pathway enrichment analysis of differentia metabolites was obtained from the KEGG database (https://www.Kegg.jp/).

### Statistical analysis.

Pearson correlation analysis was used to correlate microbiota, significant differential metabolites (SDMs), and DEGs. The transcriptome and metabolome were jointly analyzed according to different group DEGs and SDMs and mapped to the KEGG pathway.

One-way analysis of variance (ANOVA) and Duncan’s multiple-comparison test were performed on data of growth, enzyme activity, and gene expression using SPSS software (v16.0). Normal distribution and variance of the original data were checked using the Kolmogorov-Smirnov test (KS test) and Levene’s test. Differences were considered significant at a *P* value of <0.05.

### Data availability.

All the data that support the findings of the study are openly available in the National Center for Biotechnology Information (NCBI) via the accession number PRJNA937423.
