# Supplementary material for: Probiotics Mediate Intestinal Microbiome and Microbiota-Derived Metabolites Regulating the Growth and Immunity of Rainbow Trout (Oncorhynchus mykiss)
Source: Microbiol Spectr. 2023 Mar 14;11(2):e03980-22. doi: 10.1128/spectrum.03980-22 (PMC10101061; doi:10.1128/spectrum.03980-22)
Supplement: Supplemental file 1 — Supplemental material. Download spectrum.03980-22-s0001.pdf, PDF file, 0.9 MB [file spectrum.03980-22-s0001.pdf]

Supplemental Material

TABLE S1 Primers for gene expression

Gene	Forward	Reverse
Lectin	CATTACTGTTTGCTATTGGGAGGT	GGATGTAGTTATCGGGTTGCC
LYG	ATCATCGCTGGCATCATCTC	CGTCCATTCGCTCATAGGTG
HSP70b	GCTGGGCTGAATGTGCTG	GTCCTTCTTGTGTTTCCTCTTGA
IL-16	ACGAGGACTCCACCTACGACT	TCCAGCAGGCGGTTCAG
tradd	AGCAGGGCAAGTTCAGCGT	CTCCAGGTCGTCCAGACAAAA
igfbp2	CGGATGACTGGATGAACAAGG	CGGCGATGCTTTCCTCTTC
rpl35a	TTGGTTATGCGTTTCTGTCG	TGTGCTCCCGCTGGTTC
Sem1	AGGATTGGACTGGGTTGG	GCTCCGCTCTTAGTTGATTAG
siglec11	TGCGGTGGGTGTAGTGA	GCATCCGTCTGTTGGTC
mrc2	GACTCGTGGGCGGATGA	GGATGCCAGTAAGTGAAGATGA
ADAM17	TGAGTGCCCAAACCCAGAC	GGTGCCATTTTTGTCCCG
Pygmb	AATGGCTGGCAGGTAGAGG	AGGTTGAAATCACAGGGGG
itga5	GTCACGACACCCGCATCTAC	CCGTCCTATCGCCATAAACA
slc27a4	CACAGAGGTGGAGGGAACA	TCTTTGAGGAAGCGTAGGAA
EF1-α	TCCTCTTGGTCGTTTCGCTG	ACCCGAGGGACATCCTGTG
β-actin	TGGGGCAGTATGGCTTGTATG	CTCTGGCACCCTAATCACCTCT

Supplemental Material

TABLE S2 RNA-Seq sequencing results of the rainbow trout intestine transcriptome

Sample	Raw reads	Clean reads	Mapped reads	Mapped rate (%)
CK1	44125760	41733290	36855097	88.31
CK2	44412422	41897682	36841689	87.93
CK3	45429764	43163816	38511721	89.22
BV1	42315748	40795392	36105833	88.5
BV2	42805994	41221344	36725485	89.09
BV3	45468060	43792132	39011925	89.08

Supplemental Material

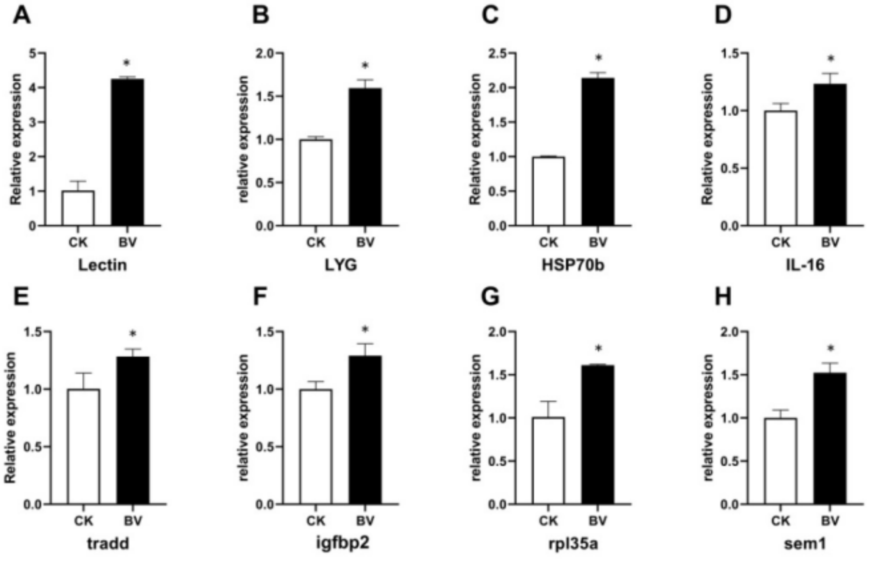

FIG S1 Validation of differential genes for *B. velezensis* MVCR2 supplementation group. Data are given as mean ± SD; Symbols of bars indicate significant differences ($P < 0.05$).

Supplemental Material

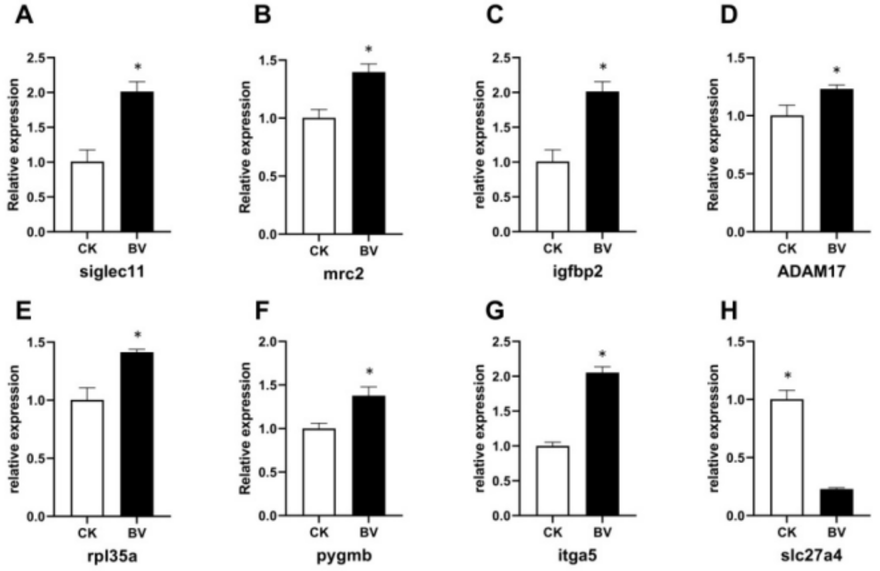

FIG S2 Validation of differential genes for *L. sakei* rMA-2 strain supplementation group. Data are given as mean ± SD; Symbols of bars indicate significant differences ($P < 0.05$).

Supplemental Material

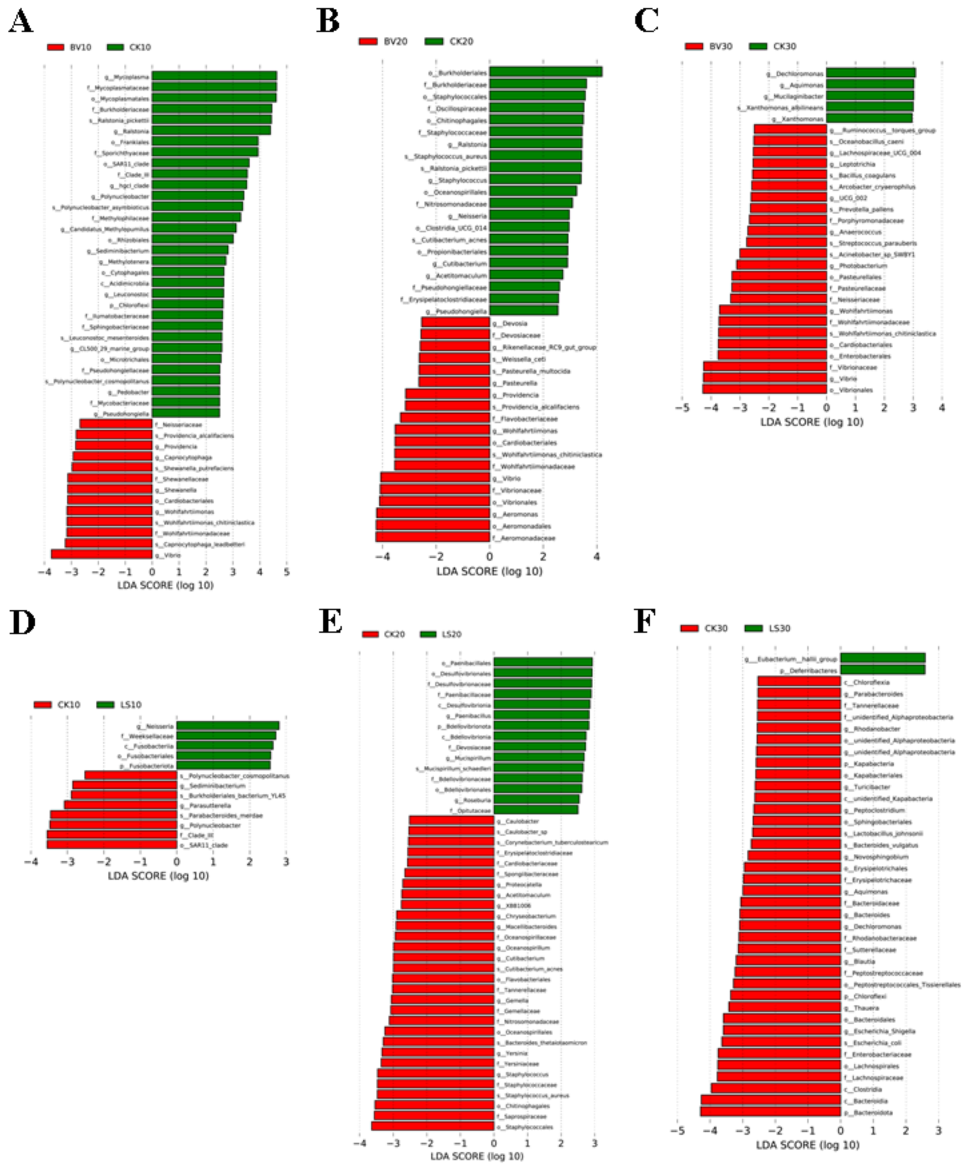

FIG S3 LEfSe analysis for differentially abundant of intestinal flora (LDA > 2.5). The color (red or green) indicated the enrichment of the taxa within the corresponding groups. The letters p, c, o, f and g represented phylum, class, order, family and genus, respectively.

Supplemental Material

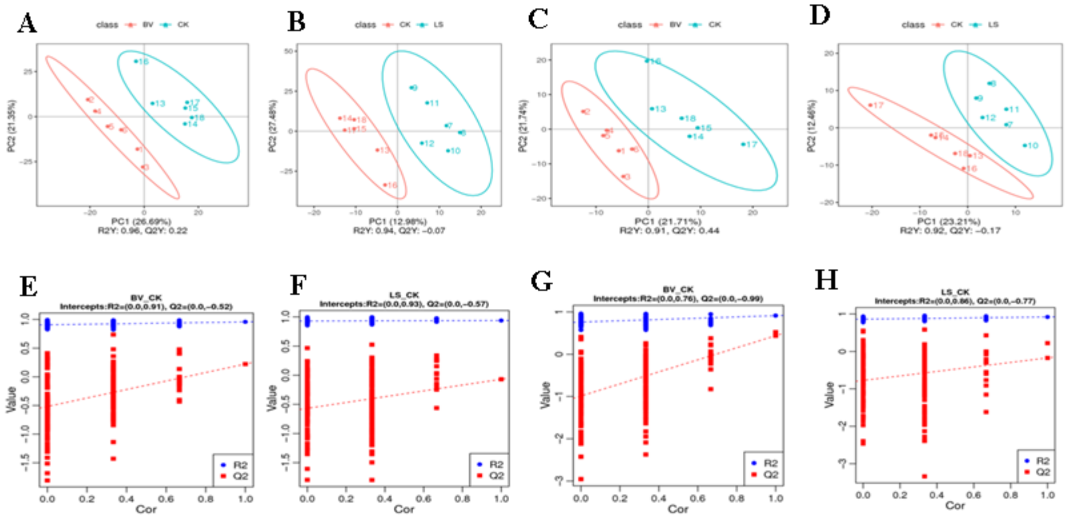

FIG S4 PLS-DA score scatter diagram and sorting verification diagram. A, B, E and F are positive modes. C, D, G and H are negative modes. A, B, C and D: PLS-DA score chart. E, F, G and H: validation diagram of PLS-DA model. PLS-DA score chart, R2Y represents the interpretation rate of the model, and Q2Y is used to evaluate the prediction ability of PLS-DA model. The abscissa is the score of the sample on the first principal component; The ordinate is the score of the sample on the second principal component.
